# Necroptosis-Related Prognostic Model for Pancreatic Carcinoma Reveals Its Invasion and Metastasis Potential through Hybrid EMT and Immune Escape

**DOI:** 10.3390/biomedicines11061738

**Published:** 2023-06-16

**Authors:** Haichuan Liu, Zhenghang Li, La Zhang, Mi Zhang, Shanshan Liu, Jianwei Wang, Changhong Yang, Qiling Peng, Chengyou Du, Ning Jiang

**Affiliations:** 1 Department of Hepatobiliary Surgery, The First Affiliated Hospital of Chongqing Medical University, Chongqing 400016, China; 2 Department of Breast and Thyroid Surgery, The First Affiliated Hospital of Chongqing Medical University, Chongqing 400016, China; 3 School of Basic Medical Science, Chongqing Medical University, Chongqing 400016, China; 4 Department of Bioinformatics, Chongqing Medical University, Chongqing 400016, China; 5 Department of Pathology, Chongqing Medical University, Chongqing 400016, China; 6 Molecular Medicine Diagnostic and Testing Center, Chongqing Medical University, Chongqing 400016, China; 7 Department of Pathology, The First Affiliated Hospital of Chongqing Medical University, Chongqing 400016, China

**Keywords:** pancreatic adenocarcinoma, prognostic model, necroptosis-related genes, invasion and metastasis, EMT, immune escape

## Abstract

Necroptosis, pro-inflammatory programmed necrosis, has been reported to exert momentous roles in pancreatic cancer (PC). Herein, the objective of this study is to construct a necroptosis-related prognostic model for detecting pancreatic cancer. In this study, the intersection between necroptosis-related genes and differentially expressed genes (DEGs) of pancreatic ductal adenocarcinoma (PDAC) was obtained based on GeneCards database, GEO database (GSE28735 and GSE15471), and verified using The Cancer Genome Atlas (TCGA). Next, a prognostic model with Cox and LASSO regression analysis, and divided the patients into high-risk and low-risk groups. Subsequently, the Kaplan–Meier (KM) survival curve and the receiver operating characteristic (ROC) curves were generated to assess the predictive ability of overall survival (OS) of PC patients. Gene ontology (GO) and Kyoto Encyclopedia of Genes and Genomes (KEGG) analyses were performed to predict the potential biofunction and possible mechanical pathways. The EMTome database and an immune analysis were applied to further explore underlying mechanism. Finally, clinical samples of PDAC patients were utilized to verify the expression of model genes via immunohistochemistry (IHC), and the normal human pancreatic ductal cell line, hTERT-HPNE as well as human pancreatic ductal carcinoma cell lines, PANC-1 and PL45, were used to identify the levels of model genes by Western blot (WB) and immunofluorescence (IF) in vitro. The results showed that 13 necroptosis-related DEGs (NRDEGs) were screened based on GEO database, and finally four of five prognostic genes, including KRT7, KRT19, IGF2BP3, CXCL5, were further identified by TCGA to successfully construct a prognostic model. Univariate and multivariate Cox analysis ultimately confirmed that this prognostic model has independent prognostic significance, KM curve suggested that the OS of low-risk group was longer than high-risk group, and the area under receiver (AUC) of ROC for 1, 3, 5 years was 0.733, 0.749 and 0.667, respectively. A GO analysis illustrated that model genes may participate in cell–cell junction, cadherin binding, cell adhesion molecule binding, and neutrophil migration and chemotaxis, while KEGG showed involvement in PI3K-Akt signaling pathway, ECMreceptor interaction, IL-17 signaling pathway, TNF signaling pathway, etc. Moreover, our results showed KRT7 and KRT19 were closely related to EMT markers, and EMTome database manifested that KRT7 and KRT19 are highly expressed in both primary and metastatic pancreatic cancer, declaring that model genes promoted invasion and metastasis potential through EMT. In addition, four model genes were positively correlated with Th2, which has been reported to take part in promoting immune escape, while model genes except CXCL5 were negatively correlated with TFH cells, indicating that model genes may participate in immunity. Additionally, IHC results showed that model genes were higher expressed in PC tissues than that in adjacent tumor tissues, and WB and IF also suggested that model genes were more highly expressed in PANC-1 and PL45 than in hTERT-HPNE. Tracing of a necroptosis-related prognostic model for pancreatic carcinoma reveals its invasion and metastasis potential through EMT and immunity. The construction of this model and the possible mechanism of necroptosis in PDAC was preliminarily explored to provide reliable new biomarkers for the early diagnosis, treatment, and prognosis for pancreatic cancer patients.

## 1. Introduction

Pancreatic cancer (PC), known as the “king of cancer”, is one of the most aggressive solid organ tumors [1]. The most common pathological type of PC is pancreatic ductal adenocarcinoma (PDAC), and approximately 350,000 people worldwide die yearly due to this cancer [2]. According to the World Health Organization, PC is projected to become the second leading cause of cancer-related mortalities by 2030, due to a five-year survival rate of nearly 10% [3]. In addition to limited treatment effects caused by the high heterogeneity and fibrosis of PC itself [1], another important reason for the high mortality of PC is the difficulty of early diagnosis due to the concealed location of pancreas, no obvious symptoms at early stage and lack of specific diagnostic biomarkers [4]. Therefore, detecting PC and its precursors early seems to be the most effective way to reduce mortality, and combining conventional chemotherapies with new therapies directly targeting the molecular changes in PC may be the most promising strategy.

Necroptosis, pro-inflammatory programmed cell necrosis independent of caspase activity, is a process of self-destruction activated by extracellular or intracellular signals especially when apoptosis is blocked [5,6]. At the molecular level, necroptosis has been identified to rely on phosphorylation of mixed lineage kinase domain-like (MLKL) induced by receptor-interacting protein kinase 3 (RIPK3) that phosphorylated MLKL translocates to the plasma membrane to cause ion influx, resulting in cell swelling and rupturing, and then the uncontrollability release of intracellular material [7,8]. Interestingly, necroptosis is proposed to be a double-edged sword throughout tumor development and progression. On the one hand, necroptosis may exert antitumor effects. Aaes et al. [9] first confirmed that necroptotic cancer cells could induce the maturation of dendritic cells, cross-priming of cytotoxic T cells, and enhance the production of IFN-γ, indicating that necroptosis is an immunogenic cell death that may stimulate adaptive immune system to exhibit anti-tumor immunity. A subsequent study pointed out that the effect of radiotherapy depends on the immune system rather than on direct induction of tumor cell death and that radiotherapy increases necroptosis through the ZBP1-MLKL pathway to release mitochondrial DNA of tumor cells, which is closely monitored by the intracellular CGAS-STING pathway then to initiate antitumor innate and adaptive immune responses [10]. On the other hand, necroptosis could restrain immune response against tumor and promote tumor growth as well as metastasis. Research has been undertaken suggesting that inhibition of necroptosis by MLKL ablation may significantly reduce lung metastasis of breast cancer [11]. The first step of the process of metastasis is that tumor cells escape from the endothelial barrier of the blood stream, and tumor cell-induced endothelial necroptosis could promote tumor cell extravasation and metastasis [12]. Treatment with trifluoperazine (TFP) induces cell death by apoptosis and necroptosis in U87MG Glioma Cells due to a coupled endoplasmatic reticulum and mitochondrial stress [13]. AURKA interacted directly with RIPK1 and RIPK3 to reduce necrosome activation, and increased expression of AURKA in pancreatic cancer tumors in patients correlated with poor overall survival, supporting the notion that AURKA may constitute a relevant therapeutic target for patients with PDAC [14]. Evidence showed that necroptosis enhanced tumor-associated macrophages (TAMs)-induced adaptive immune suppression to accelerate PDAC progression through the CXCL1 and Mincle signaling network [15]. Collectively, necroptosis has emerged as a major area of interest within cancer research. However, the exact regulatory mechanism of necroptosis in PDAC remains largely unknown.

**Figure 1:**
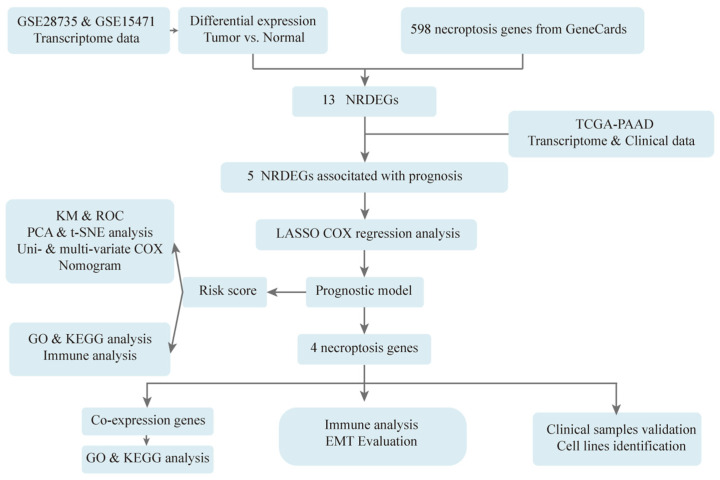
Flowchart of construction and analysis of the prognostic model. Original data of pancreatic cancer patients were downloaded from GEO, and the set of overlapping necroptosis-related genes and differentially expressed genes was screened, and further validated in TCGA. Then, LASSO COX regression analysis was applied to establish a necroptosis-related prognostic model, and univariate together with multivariate COX regression, KM survival curve analysis, ROC curve and nomogram were used to evaluate the efficiency of the risk model. In addition, GO and KEGG were performed to explore the underlying mechanism of necroptosis in PDAC. Finally, clinical samples of PDAC patients were utilized to verify the expression of model genes by IHC, and the normal human pancreatic ductal cell line, hTERT-HPNE as well as human pancreatic ductal carcinoma cell lines, PANC-1 and PL45 were used to identify the levels of model genes by WB and IF in vitro.

Therefore, in this study, as shown in Figure 1, original data of pancreatic cancer patients were downloaded from GEO, and the set of overlapping necroptosis-related genes and differentially expressed genes was screened and further validated in TCGA. Then, LASSO COX regression analysis was applied to establish a necroptosis-related prognostic model, and univariate together with multivariate COX regression, KM survival curve analysis, ROC curve and nomogram were used to evaluate the efficiency of the risk model. In addition, GO and KEGG were performed to explore the underlying mechanism of necroptosis in PDAC. Finally, clinical samples of PDAC patients were utilized to verify the expression of model genes by IHC, and the normal human pancreatic ductal cell line, hTERT-HPNE as well as human pancreatic ductal carcinoma cell lines, PANC-1 and PL45 were used to identify the levels of model genes by WB and IF in vitro. The construction of a necroptosis-related prognostic model and the possible mechanism of necroptosis in PDAC was preliminarily explored to provide reliable new biomarkers for the early diagnosis, treatment, and prognosis for PC patients.

## 2. Materials and Methods

### 2.1. Data Collection and Processing

The gene expression profiles of PC were downloaded from the GEO dataset (GSE28735, GSE15471) (https://www.ncbi.nlm.nih.gov/gds/, accessed on 11 September 2022) [16]. The datasets of PC (TCGA-PAAD), including their gene expression profiles, clinicopathological information and survival information, were downloaded from the TCGA database (https://www.cancer.gov/tcga, accessed on 11 September 2022) [17]. We screened and downloaded 598 necroptosis-related mRNA from GeneCards database (https://www.genecards.org/, accessed on 11 September 2022) [18].

### 2.2. Identification of DEGs

Due to the sample symmetry in the GEO database, we believe that the DEGs obtained from the GEO database are more representative than the TCGA database. Therefore, we obtained the DEGs from GSE28735 and GSE15471, respectively. The DESeq2 method with an adjusted *p*-value < 0.05 and |log2 fold change (FC)| > 1 setting as the threshold was employed to identify DEGs between normal and tumor samples.

### 2.3. Establishment of Risk Prognostic Model

We intersected DEGs with necroptosis-related genes to obtain 13 necroptosis-related DEGs (NRDEGs). Their differential expressions were further validated in the TCGA database. The KM plots of 12 genes were obtained via survminer package of R for visualization (version 0.4.9; R Foundation for Statistical Computing, Vienna, Austria) and survival package of R for statistical analysis of survival data (version 3.2-10), and of which 5 genes (KRT7, KRT19, CXCL5, IGF2BP3, and PKM) have prognostic significance. LASSO regression model (R package “glmnet”, version 4.1-2) was then utilized to narrow down the candidate genes and to develop the prognostic model. The risk score was calculated using the following formula: risk score = expression of Gene 1 × β1 + expression of Gene 2 × β2 + … expression of Gene n × βn, where β represents the regression coefficient of the genes in the signature.

### 2.4. Evaluation of Risk Prognostic Model

The risk score of each TCGA-PAAD sample was calculated according to the risk score calculation formula, and patients were divided into the high-risk group and the low-risk group with the median as the boundary. KM survival curves were obtained by log-rank test to compare OS between the two groups. ROC, PCA and t-SNE analyses were used to further validate the feasibility of the risk score prediction model in pancreatic cancer patients. A univariate Cox proportional hazard model was used to analyze the correlation between the prognostic model and OS, and a multivariate Cox regression analysis was used to evaluate whether the established prognostic model could serve as an independent prognostic predictor. In addition, to comprehensively assess patient survival, we constructed a nomogram using the “rms” R package, integrating different clinicopathological information, including age, gender, TMN stage, pathological stage, and risk score. To discover the link between prognostic model genes and clinical parameters, we constructed heatmaps for visualization through R package: ComplexHeatmap package (version 2.2.0).

### 2.5. Functional Enrichment Analysis

In order to further analyze the biological functions and pathways of model genes in PC, cluster Profilter R package was used to conduct enrichment analysis of two patterns, including 60 co-expressed genes of model genes and differential genes between high- and low-risk groups. GO is a gene function classification system that describes the molecular functions, cellular components, and biological processes of genes [19]. KEGG is a database that systematically analyzes gene function, linking genomic information and functional information. Adjusted *p*-value less than 0.05 was considered statistically significant [20].

### 2.6. Evaluation of EMT

The results of GO showed that model genes might participate in cell–substrate adhesion and junction, cell adhesion, cadherin binding, structural constituent of cytoskeleton, and the results of KEGG suggested that those genes were probably involved in ECM–receptor interaction, focal adhesion and cytoskeleton, which seems to be associated with EMT. Next, we also evaluated the roles of model genes on EMT. We used the corrplot R package to conduct correlation analysis between model genes and EMT-related genes mentioned in the literature [21], and then the EMTome database [22] was used to analyze model genes’ expression in primary and metastatic tumors, as well as gene mutations of these genes.

### 2.7. Immune Analysis

GO analyses showed that model genes were related to granulocyte chemotaxis, neutrophil migration and chemotaxis, while KEGG suggested that these genes were involved in the IL-17 signaling pathway, cytokine–cytokine receptor interaction and the TNF signaling pathway, illustrating that prognosis model genes were relevant to immune-microenvironment. Then, the GSVA R package (version 1.34.0) [23] was used to perform immune infiltration analysis to further discuss the effects of this prognosis model genes on immunity.

### 2.8. Hematoxylin and Eosin (H&E) Staining and Masson Staining

Clinical paraffin-embedded in 20 paired tumors and adjacent tissues of PC patients were collected from the First Affiliated Hospital of Chongqing Medical University and sliced into 3 um sections, and this study was approved by the Ethics Committee of Chongqing Medical University. Sections of PC tumors and adjacent tissues underwent dewaxing, including soaking twice in xylene for 10 min and dehydrating with ethanol in different concentrations:100% (5 min), 95% (5 min), 85% (5 min) and 75% (5 min) in sequence. Next, according to instructions, these sections were stained by using H&E reagent (Beyotime Biotechnology, Shanghai, China) and Masson staining (MXB Biotechnologies, China). A light microscope was applied to examine the morphology of tissue.

### 2.9. Immunohistochemistry Analysis

Sections were baked at 65 °C for 1.5 h until fully deparaffinized, then rehydrated with graded alcohol series and washed with PBS. Endogenous peroxidase activity was blocked by using hydrogen peroxide (3%). Antigen recovery was microwaved in citrate (pH = 6.0) buffer. Then, blocked with 5% normal goat serum for 30 min at room temperature. The remaining steps were in accordance with the kit instructions (SP-9000, OriGene, Beijing, China). Primary antibodies against KRT7 (1:50, Wuhan Boster Bio-Engineering Limited Company, Wuhan, China), KRT19 (1:50, Wuhan Boster Bio-Engineering Limited Company, Wuhan, China), IGF2BP3 (1:50, Signalway Antibody, MD, USA), and CXCL5 (1:50, Sangon Biotechnology, Shanghai, China) were added to these tissue sections, followed by incubation at 4 °C overnight. Sections were then incubated with the corresponding secondary antibody for 30 min at 37 °C. Finally, each sample is evaluated under a light microscope.

### 2.10. Cell Culture

Normal human pancreatic ductal cell, hTERT-HPNE, were purchased from BeNa Culture Collection (BNCC) of China, human pancreatic ductal carcinoma cell line, PANC-1, was purchased from Boster Bio-Engineering Limited Company of Wuhan of China, and human pancreatic ductal carcinoma cell line, PL45, was purchased from FuHeng BioLogy Company of China. Cells were maintained in Dulbecco’s Modified Eagle’s Medium (DMEM, Gibco, Shanghai, China) supplemented with 10% fetal bovine serum (FBS, Cell-Box Biological Products Trading Co., Ltd., Hong Kong, China) and 1% penicillin–streptomycin (Beyotime Biotechnology, Shanghai, China) at 37 °C in a humidified 5% CO_2_ atmosphere.

### 2.11. Western Blotting

Normal human pancreatic ductal cell, hTERT-HPNE, and human pancreatic ductal carcinoma cell lines, PANC-1 and PL45 were homogenized with a cell lysis buffer for Western blotting (Beyotime Biotechnology, Shanghai, China) comprising of 1% phenylmethanesulfonyl fluoride (PMSF, Beyotime Biotechnology, Shanghai, China). The loading buffer was mixed with supernatants. Different percentages of SDS-PAGE gels were chosen for separating total protein based on different protein molecular weights. Then, the wanted proteins were electrotransferred to polyvinylidene fluoride (PVDF) membranes at different times based on protein molecular weights. After blocking, the protein bands were incubated with relative primary antibodies including KRT7 (1:500, Sangon Biotechnology, Shanghai, China), KRT19 (1:500, Boster Bio-Engineering Limited Company, Wuhan, China), IGF2BP3 (1:500, Signalway Antibody, USA), CXCL5 (1:500, Boster Bio-Engineering Limited Company, Wuhan, China), and β-actin (1:1000, Proteintech, Wuhan, China) overnight at 4 °C. Secondary antibodies were used to incubate the membranes for 1 h. An imaging densitometer (Bio-Rad, Hercules, CA, USA) was applied to detect the densities of the bands, whose gray values were quantified by ImageJ.

### 2.12. Immunofluorescence (IF) Analysis

The coverslips of hTERT-HPNE, PANC-1 and PL45 cells were fixed, permeabilized, and blocked. The primary antibodies, including KRT7 (1:50, Sangon Biotechnology, Shanghai, China), KRT19 (1:50, Wuhan Boster Bio-Engineering Limited Company, China), IGF2BP3 (1:50, Signalway Antibody, USA), and CXCL5 (1:50, Sangon Biotechnology, China) were used at 4 °C for 12 h. Subsequently, coverslips were incubated with CoraLite594–conjugated Goat Anti-Rabbit IgG antibody (1:200, Proteintech, SA00013-4) for 1 h away from light. Ten minutes after dropping Antifade Mounting Medium with DAPI (Beyotime Biotechnology, Shanghai, China) on the coverslips and sections, visualization was accomplished with a laser scanning confocal microscope (LSCM).

### 2.13. Statistical Analysis

All data are exhibited as means ± SD (standard errors), and images were produced by GraphPad Prism software (version 6.0) (GraphPad Software Inc., San Diego, CA, USA). Comparisons between groups were conducted using Student’s *t*-test. A *p*-value of <0.05 indicated a statistically significant difference.

## 3. Results

### 3.1. Five NRDEGs Were Identified Based on GEO and TCGA Database

**Figure 2:**
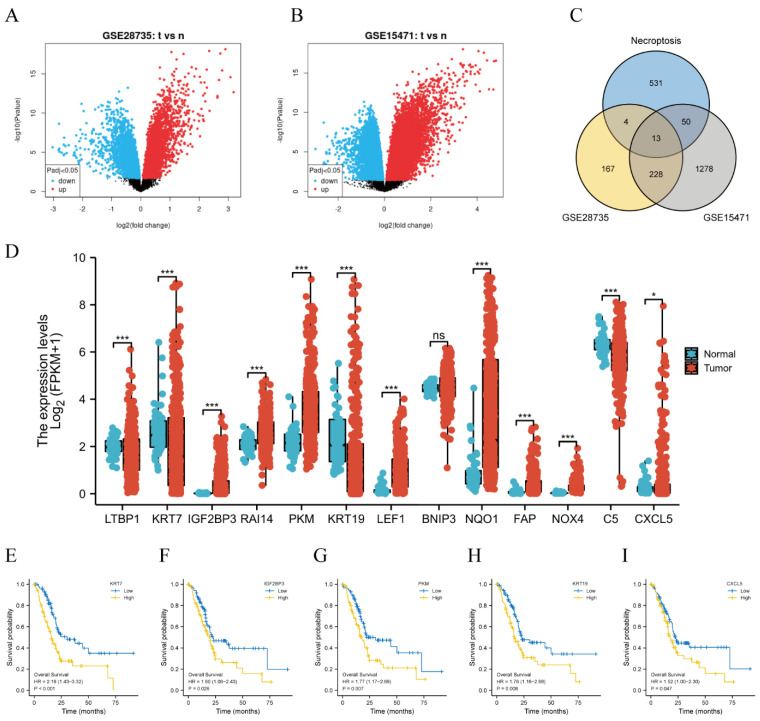
Five NRDEGs were identified based on GEO and TCGA database. (**A**) Volcano plot of differentially expressed genes from GSE28735. (**B**) Volcano plot of differentially expressed genes from GSE15471. (**C**) Venn diagram of the intersection of DEGs and necroptosis-related genes. (**D**) Differential expression of 13 NRDEGs in the TCGA database. * means *p* < 0.05, *** means *p* < 0.001 and ns means no sense. (**E**–**I**) Kaplan–Meier curves of NRDEGs: (**E**) KRT7 (*p* < 0.001), (**F**) IGF2BP3 (*p* = 0.026), (**G**) PKM (*p* = 0.007), (**H**) KRT19 (*p* = 0.008), (**I**) CXCL5 (*p* = 0.047).

First, 412 DEGs were obtained from GSE28735, and 1569 DEGs were obtained from GSE15471 in the GEO database, as shown in Figure 2A,B. The intersection of the above DEGs and necroptosis-related genes was built to gain 13 necroptosis-related DEGs (NRDEGs), including LTBP1, KRT7, IGF2BP3, RAI14, PKM, KRT19, LEF1, BNIP3, NQO1, FAP, NOX4, C5, and CXCL5 (Figure 2C). Next, differential expression was further validated in the TCGA database, except for BNIP3 (Figure 2D), other genes were differentially expressed. Subsequently, the KM plots of 12 genes were drawn through the survminer package (version 0.4.9) and survival package (version 3.2-10), as displayed in Figure 2E–I and Supplementary Figure S1, five genes including KRT7, KRT19, CXCL5, IGF2BP3, and PKM, had prognostic significances.

### 3.2. A Necroptosis-Related Risk Prognostic Model Was Successfully Constructed

**Figure 3:**
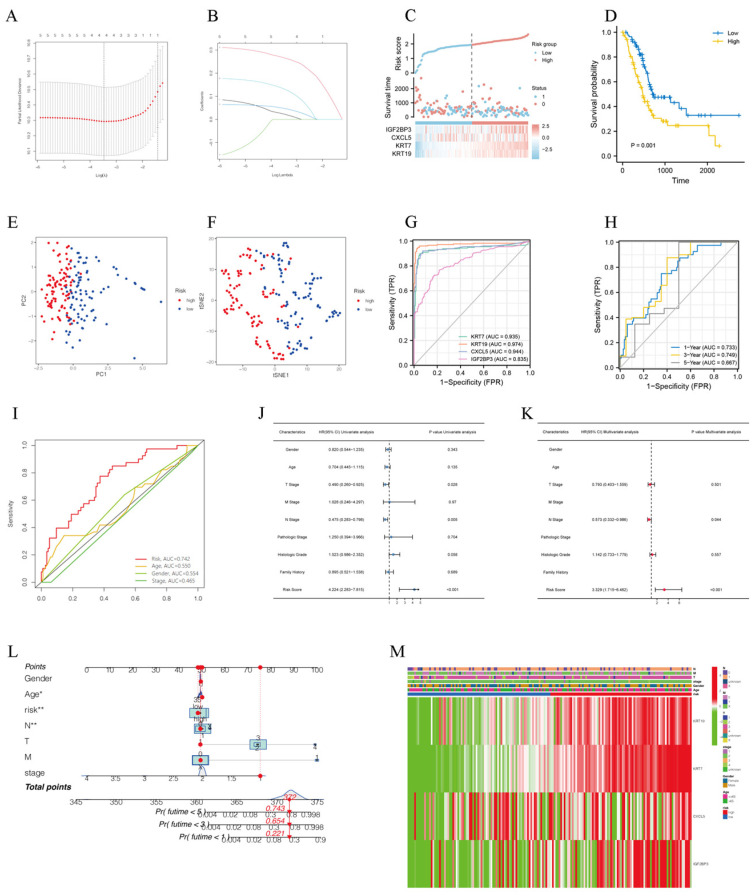
A necroptosis-related risk prognostic model was successfully constructed. (**A**) Ten-time cross-validation for tuning parameter selection in the LASSO model. (**B**) LASSO coefficient profiles. (**C**) The risk score, survival status, and heat map of model genes in patients with PC. (**D**) Kaplan–Meier curves showed that high-risk group had a worse prognosis than the low-risk group. (**E**,**F**) PCA and t-SNE visualization of low- and high-risk clusters. (**G**) ROC curves of KRT7, KRT19, CXCL5 and IGF2BP2. (**H**) ROC curves of prognostic model for 1, 3, 5 years, (**I**) ROC curves of risk score and clinicopathological characteristics. (**J**,**K**) Univariate and multivariate Cox regression analysis of clinicopathological features of PC associated with OS. (**L**) Clinical characteristics and prognostic model were used to establish a predictive nomogram. * means *p* < 0.05, ** means *p* < 0.01 (**M**) Heatmap of model genes and clinical parameters.

Lasso analysis was performed to screen four of five NRDEGs, including KRT7, KRT19, CXCL5, and IGF2BP3, to construct the best risk prognosis model with a penalty coefficient of 4 (Figure 3A–C). The corresponding regression coefficients β1–β4 were obtained with values of 0.235, 0.004, 0.033, and 0.101, respectively. Risk score of each patient can be calculated by the expression of the four genes and the corresponding regression coefficient, and the formula was as follows: Risk score = EXP KRT7 × 0.235 + EXP KRT19 × 0.004 + EXP CXCL5 × 0.033 + EXP IGF2BP3 × 0.101. According to risk scores, the patients were divided into high-risk and low-risk groups by median split. The results of KM curves showed that the high-risk group had a worse prognosis than the low-risk group (Figure 3D). Moreover, principal component analysis (PCA) and t-distributed stochastic neighbor embedding (t-SNE) analysis were performed, and as shown in Figure 3E,F, PC patients were divided into different risk groups with a relatively clear resolution. Diagnostic ROC on four genes by pROC R package showed that AUC values of the four genes were 0.935, 0.974, 0.944, and 0.835, respectively (Figure 3G), indicating that model genes screened have a high-performance diagnostic value for PC patients. Time-dependent ROC curves were employed to assess the accuracy of established models for predicting OS in PC patients and showed that AUC values of 1, 3, and 5 years were 0.733, 0.749, and 0.667, respectively (Figure 3H). The ROC of clinical parameter models and risk score suggested that compared to age, gender, and stage, the risk score has higher sensitivity and specific predictive performance (Figure 3I). Univariate Cox regression analysis elucidated that risk score, T stage, N stage and histology grade were prognostic predictors in TCGA-PAAD, but not gender, age, M stage, pathologic stage and family history (Figure 3J). More importantly, the risk score was also observed to be the independent predictor in multivariate Cox regression analysis (Figure 3K). Clinical characteristics and prognostic model were used to establish a predictive nomogram for predicting the prognostic survival probability of PC patients at 1, 3, and 5 years (Figure 3L). A heatmap revealed the correlations between model genes and clinical parameters (Figure 3M).

### 3.3. GO and KEGG Analysis Suggested That Model Genes Were Associated with Cell Adhesion and Immunity

**Figure 4:**
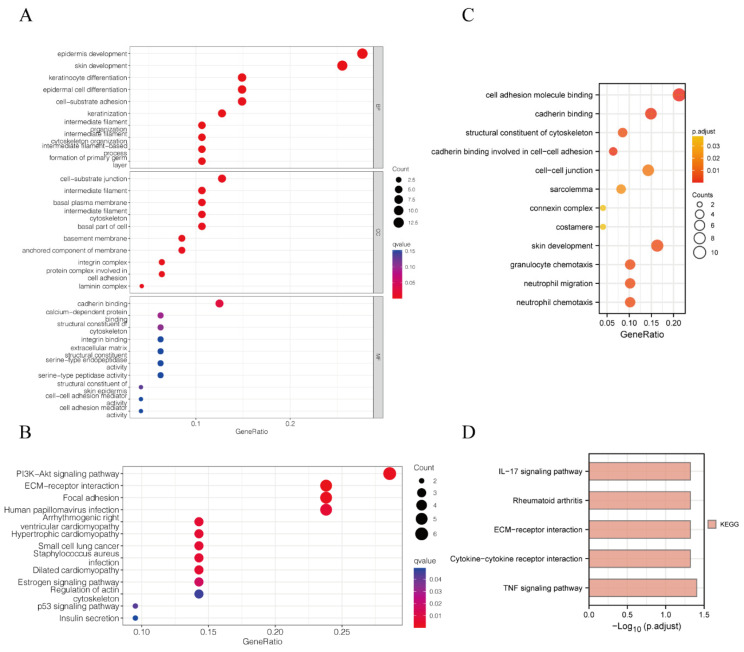
GO and KEGG analysis suggested that model genes were associated with cell adhesion and immunity. (**A**) GO analysis of the differential genes between the high- and low-risk groups. (**B**) KEGG analysis of the differential genes between the high- and low-risk groups. (**C**) GO analysis of the model genes and their co-expressed genes. (**D**) KEGG analysis of the model genes and their co-expressed genes.

In order to explore the molecular functions and pathway mechanisms of model genes, we first performed GO/KEGG analysis on the differential genes between the high- and low-risk groups, and the results showed PI3K-Akt signaling pathway, ECM–receptor interaction, focal adhesion, and cytoskeleton in the KEGG enrichment analysis and the molecular functional modules involved in cell–substrate adhesion, cell–substrate junction, cell adhesion, and cadherin binding in the GO enrichment analysis (Figure 4A,B). In addition, we further performed GO/KEGG analysis on model genes and their co-expressed genes, and the results showed IL-17 signaling pathway, ECM–receptor interaction, cytokine–cytokine receptor interaction and the TNF signaling pathway in the KEGG enrichment analysis and granulocyte chemotaxis, neutrophil migration and chemotaxis, cell adhesion molecule binding, structural constituent of the cytoskeleton, and acadherin binding involved in cell−cell adhesion in the GO enrichment analysis (Figure 4C,D). Interestingly, two different patterns of GO/KEGG analyses led to almost the same conclusion: our model genes were associated with adhesion and metastasis as well as infiltration of PC immune cells.

### 3.4. Model Genes Were Involved in EMT of PC

**Figure 5:**
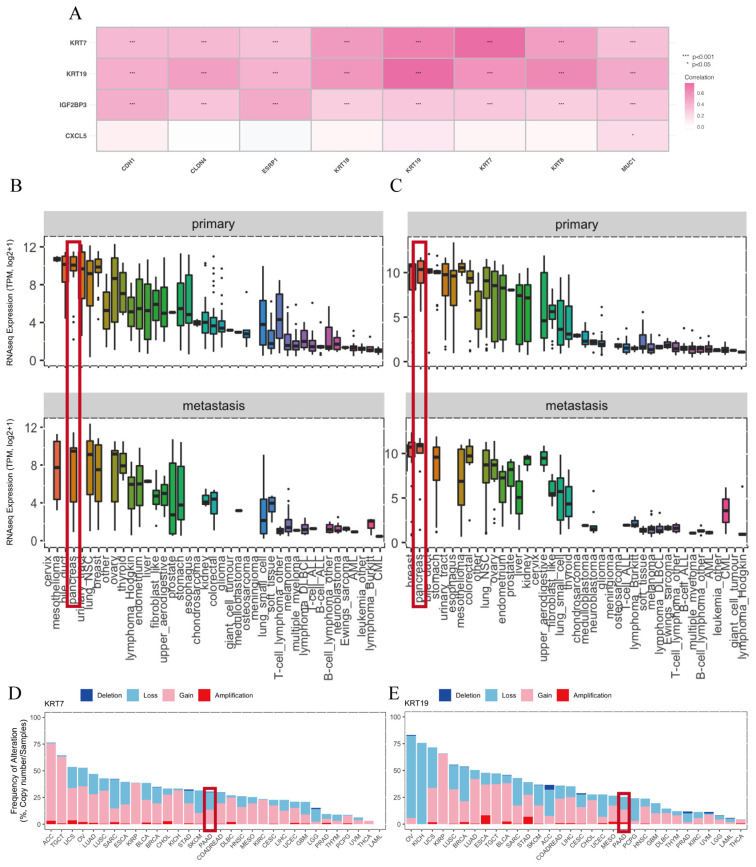
Model genes were involved in EMT of PC. (**A**) The correlation heat map between model genes and EMT-related genes. (**B**) KRT7 expression in primary as well as metastatic tumors in the EMTome database. (**C**) KRT19 expression in primary as well as metastatic tumors in EMTome database. (**D**) Gene mutations of KRT7 in various cancers in EMTome database. (**E**) Gene mutations of KRT19 in various cancers in EMTome database.

The results of GO showed that model genes might participate in cell–substrate adhesion and junction, cell adhesion, cadherin binding, structural constituent of cytoskeleton, and while the results of KEGG suggested that those genes were probably involved in ECM–receptor interaction, focal adhesion and the cytoskeleton, which seems to be probably associated with epithelial–mesenchymal transition (EMT). EMT is a cellular process during which cells lose their epithelial characteristics and gain mesenchymal characteristics, allowing them to migrate and invade the underlying mesenchyme more efficiently. Next, the correlation analysis demonstrated that KRT7, KRT19 and IGF2BP3 had strong correlations with EMT-related genes, and CXCL5 only showed weak correlations (Figure 5A). The EMTome database was used to further excavate the relationship between model genes and EMT, the data showed that model genes including KRT7 as well as KRT19, were highly expressed in both primary and metastatic PDAC (Figure 5B,C). In addition, the EMTome database further exhibited that KRT7 and KRT19 had gene mutations, including deletion, loss, gain, and amplification in PC (Figure 5D). The above results indicated that model genes affect the migration and invasion of PC most likely through EMT.

### 3.5. Model Genes Regulated Immunity of PC

**Figure 6:**
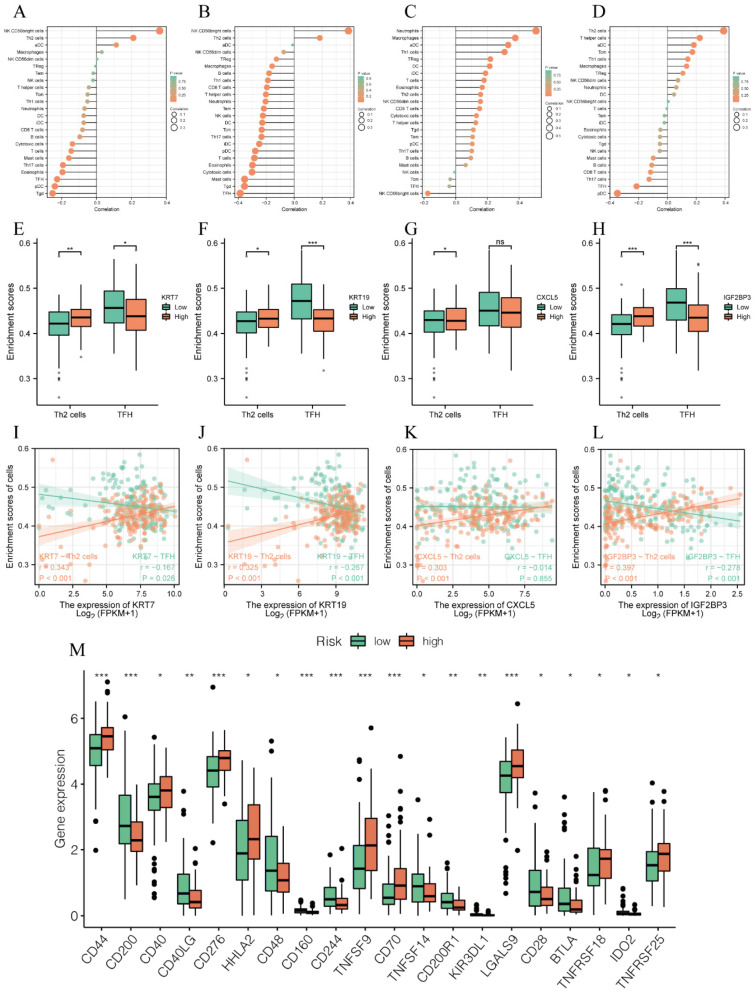
Model genes regulated immunity of PC. (**A**–**D**) The correlation of model genes with various immune cells: (**A**) KRT7, (**B**) KRT19, (**C**) CXCL5, (**D**) IGF2BP3. (**E**–**H**) Histogram of correlation between model genes and immune cells (Th2, TFH cells). (**I**–**L**) Scatter plot of correlation between model genes and immune cells (Th2, TFH cells). (**M**) Expressions of immune checkpoints between the high- and low-risk groups. * means *p* < 0.05, ** means *p* < 0.01, *** means *p* < 0.001 and ns means no sense.

According to the enrichment analysis results, the model genes are also related to cellular immunity, so immune analysis was performed to conduct an appraisal of roles of model genes on immunity. As exhibited in Figure 6A–D, there were greater or fewer correlations between model genes and various immune cells. In particular, all four genes were positively related to T helper 2 (Th2) cells, while model genes except for CXCL5 were negatively correlated with follicular helper T cells (TFH cells) (Figure 6E–L). In addition, assessments of immune checkpoints between high- and low-risk groups illustrated that CD44, CD40, CD276, CD70, TNFSF9, and LGALS9 were strongly positively correlated with our prognostic model genes (Figure 6M). The results from this section indicated that model genes might regulate immunity to affect the prognosis of PC.

### 3.6. Model Genes Were Upregulated in Clinical Pancreatic Carcinoma Samples

**Figure 7:**
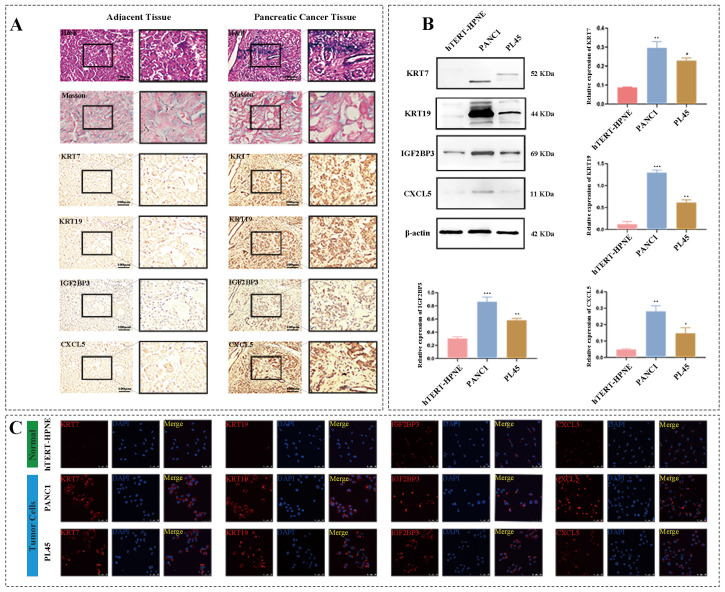
Model genes were upregulated in clinical PC samples and PC cell lines. (**A**) HE, Masson and IHC staining of model genes including KRT7, KRT19, IGF2BP3 and CXCL5 in clinical PC tissues and corresponding adjacent tumor tissues. (**B**) Western blot and histograms of model genes in hTERT-HPNE, PANC-1 and PL45. (**C**) Immunofluorescence (×400) of model genes in hTERT-HPNE, PANC-1 and PL45. hTERT-HPNE is normal human pancreatic ductal cell line, and PANC-1 and PL45 are human pancreatic ductal carcinoma cell lines. * *p* < 0.05, ** *p* < 0.01, *** *p* < 0.001 vs. hTERT-HPNE.

To further identify the reliability of model genes, clinical PC tissues and corresponding adjacent tumor tissues from First Affiliated Hospital of Chongqing Medical University were collected to validate the levels of model genes. H&E staining displayed that tumor cells were arranged as lumen-like structures with moderate to severe nuclear atypia (Figure 7A) and inflammatory cells were infiltrated in PC stroma, Masson staining showed that fibrosis of tumor tissue is more obvious than that in adjacent tissue (Figure 7A), and IHC staining revealed that the expressions of four model genes including KRT7, KRT19, IGF2BP3 and CXCL5 were all higher in tumor tissues than those in adjacent tissues (Figure 7A), which were consistent with the results of our bioinformatics analysis.

### 3.7. Model Genes Were Upregulated in Human Pancreatic Ductal Carcinoma Cells In Vitro

Subsequently, normal human pancreatic ductal cell line, hTERT-HPNE as well as human pancreatic ductal carcinoma cell lines, PANC-1, and PL45 were used to further detect the levels of model genes in vitro. Western blot results showed that, compared to hTERT-HPNE, model genes including KRT7, KRT19, IGF2BP3, and CXCL5, were significantly increased in human pancreatic ductal carcinoma cell lines, PANC-1 and PL45 (Figure 7B). Consistent with the results of WB, immunofluorescence results also suggested that the fluorescence intensity of model genes in PANC-1 and PL45 was brighter than that in hTERT-HPNE (Figure 7C). The above results indicated that model genes were upregulated in human pancreatic ductal carcinoma cells in vitro.

## 4. Discussion

Pancreatic cancer is one of the most aggressive digestive system tumors, with extremely high malignancy. Due to a lack of early diagnosis and limited traditional treatments, PC has a very poor prognosis. Thus, it is particularly important to urgently identify efficient biomarkers with excellent specificity and sensitivity for early detection and prognosis prediction for PC patients. Growing evidence suggests that necroptosis promotes the pathophysiological process of pancreatic cancer and is closely related to its invasion and metastasis [24,25]. In order to explore the possible mechanism of necroptosis in PC, in this study, original data of pancreatic cancer patients were downloaded from the GEO database, and the set of overlapping necroptosis-related genes and differentially expressed genes was screened, and further validated in the TCGA database. Then, LASSO COX regression analysis was applied to establish a necroptosis-related prognostic model, which included four genes, KRT7, KRT19, IGF2BP3 and CXCL5, and univariate analysis, together with multivariate COX regression, KM survival curve analysis, ROC curve and nomogram were used to evaluate the efficiency of the risk model. Subsequently, IHC and HE staining were performed to verify the expression of four genes (KRT7, KRT19, IGF2BP3 and CXCL5) in clinical PC tissues and corresponding adjacent tissues, and WB and IF also suggested that model genes were higher expressed in PANC-1 and PL45 than in hTERT-HPNE. More importantly, the results of GO showed that model genes might participate in cell–substrate adhesion, cell–substrate junction, cell adhesion, cadherin binding, granulocyte chemotaxis, neutrophil migration and chemotaxis, cell adhesion molecule binding, structural constituent of cytoskeleton, cadherin binding involved in cell–cell adhesion, and the results of KEGG suggested that those genes were probably involved in the PI3K-Akt signaling pathway, ECM–receptor interaction, focal adhesion and cytoskeleton, indicating that model genes affect the prognosis of PC most likely through EMT and immune escape.

In these genes, KRT7 and KRT19 are members of the keratin gene family; the former is a type II keratin composed of alkaline or neutral protein, while the latter is a type I cytokeratin by paired keratin consisting of an acidic protein chain. KRT7 is positively expressed in most epithelial cells, such as lung and breast, and it is currently mainly used in the diagnosis and differential diagnosis of breast cancer, lung cancer and gastrointestinal adenocarcinoma [26]. Chen et al. [27] found that mRNA stability and translation efficiency of KRT7 regulated by N6 -Methyladenosine may affect the induction of lung metastasis of breast cancer through transcriptome and transcriptome analysis of epithelial cells. Huang et al. [28] illustrated that long non-coding antisense RNA of KRT7 (KRT7-AS) upregulated the expression of KRT7 to accelerate the progress of gastric cancers. Moreover, a new study manifested that fusobacterium nucleatum modulated KRT7-AS and KRT7 through the NF-κB pathway to promote colorectal cancer cell migration and metastasis [29]. In our study, the expression of KRT7 increased in PDAC, and patients with high expression of KRT7 had a worse prognosis. Furthermore, KRT19 was also a widely used biomarker positively expressed in normal epithelial tissues such as the glandular epithelium of endometrium and bile duct epithelium. A report suggested that a high level of KRT19 showed the strongest correlation with increased tumor size and metastasis, and knockdown of KRT19 inhibited invasion of hepatocellular carcinoma [30]. In the present study, the level of KRT19 was also increased in PDAC, and its high level was associated with the worse prognosis of patients. In addition, CXCL5, which is a member of the CXC subfamily of chemokines, binds G-protein-coupled receptor chemokine (CXC motif) receptor 2 to recruit neutrophils to promote angiogenesis and remodel connective tissue [31]. Ando et al. [24] discovered that compared with the control group and apoptosis group, the expression of CXCL5 increased in necroptosis, its receptor CXCR2 was upregulated in PC and inhibition of CXCR2 suppressed necroptosis-induced migration and metastasis of PC cells. Thus, it is reasonable to suspect that CXCL5 released by necroptosis, probably through CXCR2, promotes migration and metastasis. Moreover, with regard to IGF2BP3, the last gene of this prognosis model, we interestingly found that it cannot be detected in most normal tissues, but it is highly expressed in embryos and different types of tumors, including PC [32,33,34]. Huang et al. [35] reported that knockdown of IGF2BPs inhibited proliferation, migration, and invasion of HeLa and HepG2 cells, and pointed out that IGF2BPs may be served as m6A readers in post-transcriptional gene regulation and cancer biology. IGF2BP3 promotes cell adhesion and invasion pseudopodia formation in HeLa cells through binding to 3’-UTR of CD44 mRNA to maintain its stability, and CD44 is considered necessary for the formation of invasive pseudopodia [36]. In our study, CXCL5 and IGF2BP3 were upregulated in PDAC, and their high expressions were associated with poor survival. Our results showed that a risk model was successfully constructed via LASSO analysis, and univariate and multivariate Cox analysis ulteriorly confirmed that this prognostic model has independent prognostic significance, KM curve suggested that the OS of the low-risk group was longer than the high-risk group, and the area under receiver (AUC) of ROC for 1, 3, 5 years was 0.733, 0.749 and 0.667, respectively, indicating that the prognosis model based on the above four genes was excellent effectiveness at predicting prognosis of PDAC patients.

Subsequently, to determine the underlying mechanism of these model genes, GO and KEGG analyses were performed. The results of GO showed that the model genes might participate in cell–substrate adhesion and junction, cell adhesion, cadherin binding, structural constituent of cytoskeleton, and the results of KEGG suggested that those genes were probably involved in ECM–receptor interaction, focal adhesion and cytoskeleton, which seems to be probably associated with EMT. Next, the EMTome database was used to excavate the relationship between model genes and EMT; the data showed that model genes except CXCL5 showed correlations with EMT-related genes, and KRT7, as well as KRT19 were highly expressed in both primary and metastatic PDAC, indicating that model genes affect the prognosis of PDAC most likely through EMT. It was found that EMT is not a binary process as a fully epithelialized state or completely mesenchymal state (C-EMT), and a hybridization state is situated between the two, known as “hybrid EMT” or “partial EMT” (p-EMT) [21,37]. Interestingly, cancer cells in the hybrid EMT state are more likely to invade and metastasize [37]. Nicole et al. [21] applied RNA sequencing to discover that in pancreatic cancer, the expressions of KRT7 and KRT19 were higher in p-EMT than in complete EMT, and identified highly expressed of KRT7 and KRT19 were more conducive to the hybridization of EMT. Furthermore, in contrast to C-EMT, pancreatic cancer cells in p-EMT were more likely to maintain cell-to-cell contact to invade adjacent tissue as a group rather than as a single cell [21]. These studies are consistent with the conclusion of our study that high expressions of KRT7 and KRT19 promote the invasion and metastasis of PDAC, probably through EMT. Moreover, Mao et al. [38] reported that the level of CXCL5 was elevated in gastric cancer, which was positively correlated with lymphatic metastasis and tumor differentiation, while recombinant human CXCL5 (rhCXCL5) could induce gastric cancer cell epithelial–mesenchymal transition (EMT) by activating the ERK pathway to enhance the migratory and invasive capacities of tumor cells. Additionally, Hanane Ennajdaoui et al. [39] highlighted that the loss of IGF2BP3 reduced the invasiveness of PDAC cells and remodeled local adhesion junctions, suggesting that IGF2BP3 was also involved in EMT. Based on the existing literature and our experimental results, we hypothesized that KRT7 and KRT19 are involved in EMT as epithelial components, while CXCL5 and IGF2BP3 act as regulatory factors to regulate EMT, thereby promoting the invasion and metastasis of pancreatic cancer.

**Figure 8:**
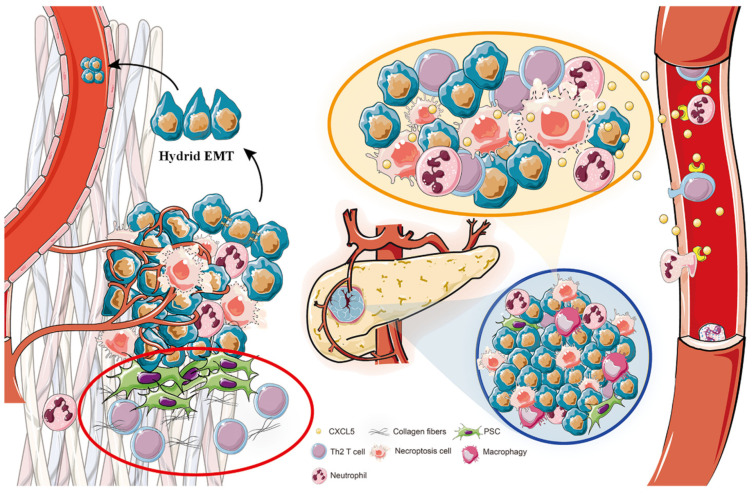
Prognostic model genes for PC reveals its invasion and metastasis potential through hybrid EMT and immune escape. Hypoxic pancreatic cancer cells are more likely to suffer necroptosis to induce inflammation and immune responses in tumor tissues, which further enhance fibrosis and heterogeneity of tumor, thus the fibrotic and inflammatory circumstance together with immune evasion induced by Th2 assist PC cells to escape from immune elimination. Furthermore, our model genes promote tumor invasion and metastasis by promoting EMT especially hybrid EMT of PC cells that KRT7 and KRT19 are involved in EMT as epithelial components, while CXCL5 and IGF2BP3 act as regulatory factors to regulate EMT. Ultimately, the above effects together promote tumor invasion and metastasis.

Furthermore, GO analyses showed that model genes were related to granulocyte chemotaxis, neutrophil migration and chemotaxis, while KEGG suggested that these genes were involved in the IL-17 signaling pathway, cytokine–cytokine receptor interaction, and TNF signaling pathway, which have been shown to play roles in the immune microenvironment of tumors [40,41,42,43], illustrating that prognosis model genes were relevant to immune microenvironment. Then, immune analysis was utilized to reveal that all four genes of risk model were positively correlated with Th2, while model genes except for CXCL5, were negatively correlated with TFH cells. Th2 is a T-cell subset secreting Th2-type cytokines (such as IL-4, IL-5, IL-10, and IL-13), differentiated from Th0 cells, and Th0 could be converted into different lineages, such as Th1 and, Th2 [44]. Under normal circumstances, Th1 cells and Th2 cells maintain a relative balance, but in tumors, the Th1/Th2 ratio loses control, with Th2 usually sustaining a high level to induce inflammation and promote tumor growth, called cancer-related inflammation [45]. With regard to PC, Th2 cells release IL-5 and IL-13 to elevate extracellular matrix deposition and affect the development of M2 macrophages to promote fibrosis of tumor tissue which is the main causes of drug delivery difficulties as well as immunosuppression [46]. Our model genes were all positively correlated with a high Th2 level, indicating that model genes may promote immune escape through Th2 cells to promote PC progress. Furthermore, TFH cells played antitumor roles in various malignant solid tumors, meaning a favorable prognosis, such as PDAC. It has been affirmed that TFH cells recruited CD8 T cells and B cells by secreting CXCL13 and facilitated the maturation of B cells into antibody-producing plasmocytes by secreting interleukin 21 (IL-21), thereby affecting tumor microenvironment [47]. In addition, our results showed immune checkpoints, especially including CD44, CD40, CD276, CD70, TNFSF9, and LGALS9, were strongly positively correlated with our prognostic model, elucidating that these immune checkpoints are likely to affect the progress of PC. More importantly, one unexpected finding was the extent to which necroptosis released CXCL5 contributing to inflammation to accelerate cancer cell migration and invasion in PC [24]. Necroptosis served as a pro-inflammatory cell death mode to further aggravate inflammation, which may activate pancreatic stellate cells (PSCs) to secrete collagen fibers to facilitate fibrosis of PC [48]. Inflammation and the fibrotic tumor micro-environment may assist PC cells in evading immune clearance and promote malignant progression and metastatic spread to distant organs [49,50]. Therefore, combining the above literature and determining the detailed molecular mechanisms strengthened the hypothesis, as displayed in Figure 8. On the one hand, hypoxic pancreatic cancer cells are more likely to suffer necroptosis to induce inflammation and immune responses in tumor tissues [10,51], which further enhance fibrosis [48] and the heterogeneity of the tumor; thus, the fibrotic and inflammatory circumstance together with immune evasion induced by Th2 [45], helps PC cells to escape from immune elimination. On the other hand, our model genes promote tumor invasion and metastasis by promoting EMT especially hybrid EMT of PC cells [37]. KRT7 and KRT19 are involved in EMT as epithelial components, while CXCL5 and IGF2BP3 act as regulatory factors to regulate EMT. Ultimately, the above effects act together to promote tumor invasion and metastasis.

## 5. Conclusions

In conclusion, in this study, the construction of this model an efficient necroptosis-related prognosis model was established to effectively assess the diagnosis and prognosis value, and provided preliminary mechanistic speculation on its potential involvement in the tumor microenvironment (TME) and immune processes, providing reliable new biomarkers for the early diagnosis, treatment, and prognosis for pancreatic cancer patients. However, our study has some limitations. Firstly, we only collected 20 pairs of clinical samples and performed validation of the model gene expression, but large-scale clinical sample validation on the diagnostic and prognostic efficacy of model genes was not conducted. Secondly, we did not conduct in-depth mechanistic investigations through molecular biology experiments. Thirdly, we did not validate the model genes through in vitro or animal experiments. In future experiments, we will further perform the relevant research to provide evidence for the clinical application of this model and conduct in-depth investigations into the mechanisms of model genes.

## Data Availability

Only publicly available data were used in this study, and data sources and handling of these data are described in the Materials and Methods and in the Supplementary Materials. Further information is available from the corresponding author upon request.

## Supplementary Materials

The following supporting information can be downloaded at: https://www.mdpi.com/article/10.3390/biomedicines11061738/s1, Figure S1: Kaplan-Meier curves of NRDEGs.
