# Supplementary material for: Necroptosis-Related Prognostic Model for Pancreatic Carcinoma Reveals Its Invasion and Metastasis Potential through Hybrid EMT and Immune Escape
Source: Biomedicines. 2023 Jun 16;11(6):1738. doi: 10.3390/biomedicines11061738 (PMC10296367; doi:10.3390/biomedicines11061738)
Supplement: Supplementary file 1 [file biomedicines-11-01738-s001.zip › biomedicines-2381288-supplementary.pdf]

Supplementary Materials

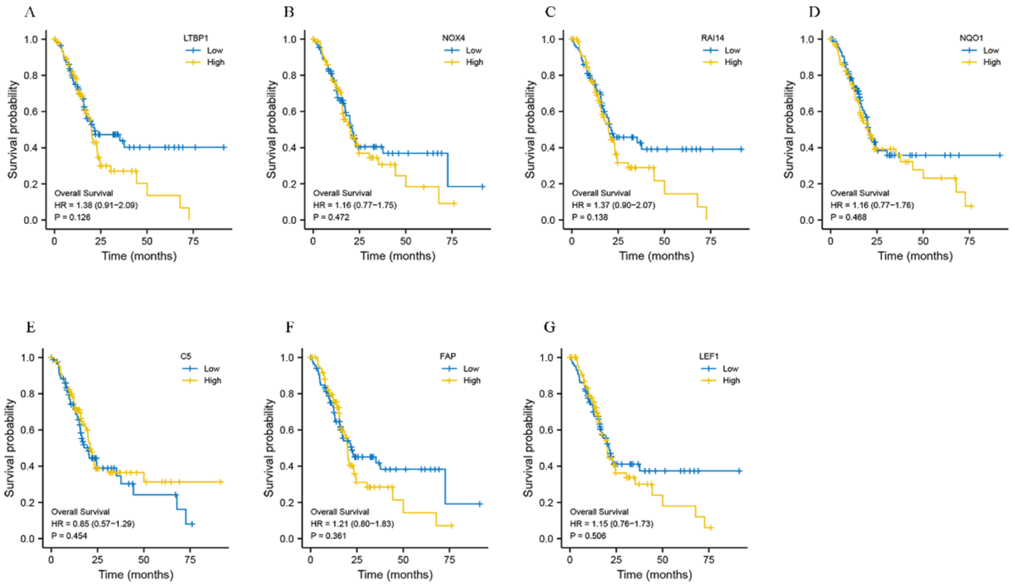

Figure S1. Kaplan-Meier curves of NRDEGs. (**A**) Kaplan-Meier curves of LTBP1 ($p > 0.05$), (**B**) Kaplan-Meier curves of NOX4 ($p > 0.05$), (**C**) Kaplan-Meier curves of RAI14 ($p > 0.05$), (**D**) Kaplan-Meier curves of NQO1 ($p > 0.05$), (**E**) Kaplan-Meier curves of C5($p > 0.05$), (**F**) Kaplan-Meier curves of FAP ($p > 0.05$), (**G**) Kaplan-Meier curves of LEF1($p > 0.05$).
